# Induction of Hypergammaglobulinemia and Autoantibodies by *Salmonella* Infection in MyD88-Deficient Mice

**DOI:** 10.3389/fimmu.2018.01384

**Published:** 2018-06-20

**Authors:** Jincy M. Issac, Yassir A. Mohamed, Ghada Hassan Bashir, Ashraf Al-Sbiei, Walter Conca, Taj A. Khan, Asif Iqbal, Gabriela Riemekasten, Katja Bieber, Ralf J. Ludwig, Otavio Cabral-Marques, Maria J. Fernandez-Cabezudo, Basel K. al-Ramadi

**Affiliations:** ^1^Department of Medical Microbiology and Immunology, College of Medicine and Health Sciences, United Arab Emirates University, Al Ain, United Arab Emirates; ^2^Department of Biochemistry, College of Medicine and Health Sciences, United Arab Emirates University, Al Ain, United Arab Emirates; ^3^Department of Internal Medicine, College of Medicine and Health Sciences, United Arab Emirates University, Al Ain, United Arab Emirates; ^4^Department of Microbiology, Kohat University of Science and Technology, Kohat, Pakistan; ^5^Department of Pharmacology, University of Illinois at Chicago, Chicago, IL, United States; ^6^Department of Rheumatology, University of Lübeck, Lübeck, Germany; ^7^Lübeck Institute of Experimental Dermatology, University of Lübeck, Lübeck, Germany

**Keywords:** MyD88 deficiency, autoantibodies, *Salmonella typhiumrium*, hypergammaglobulinemia, Tfh cells

## Abstract

Growing evidence indicates a link between persistent infections and the development of autoimmune diseases. For instance, the inability to control *Salmonella* infection due to defective toll-like receptor (TLR)/myeloid differentiation primary response 88 (MyD88) signaling has linked the development of persistent infections to a breakdown in B cell tolerance. However, the extent of immune dysregulation in the absence of TLR-MyD88 signaling remains poorly characterized. Here, we show that MyD88^−/−^ mice are unable to eliminate attenuated *Salmonella enterica* serovar Typhimurium, even when challenged with a low-dose inoculum (200 CFUs/mouse), developing a persistent and progressive infection when compared to wild-type (MyD88^+/+^) animals. The splenic niche of MyD88^−/−^ mice revealed increased counts of activated, Sca-1-positive, myeloid subpopulations highly expressing BAFF during persistent *Salmonella* infection. Likewise, the T cell compartment of *Salmonella*-infected MyD88^−/−^ mice showed increased levels of CD4^+^ and CD8^+^ T cells expressing Sca-1 and CD25 and producing elevated amounts of IL-4, IL-10, and IL-21 in response to CD3/CD28 stimulation. This was associated with increased Tfh cell differentiation and the presence of CD4^+^ T cells co-expressing IFN-γ/IL-4 and IFN-γ/IL-10. Noteworthy, infected MyD88^−/−^ mice had enhanced serum titers of both anti-*Salmonella* antibodies as well as autoantibodies directed against double-stranded DNA, thyroglobulin, and IgG rheumatoid factor, positive nuclear staining with HEp-2 cells, and immune complex deposition in the kidneys of MyD88^−/−^ mice infected with live but not heat-killed *Salmonella*. Infection with other microorganisms (*Acinetobacter baumanii, Streptococcus agalactiae*, or *Escherichia coli*) was unable to trigger the autoimmune phenomenon. Our findings suggest that dysregulation of the immune response in the absence of MyD88 is pathogen-dependent and highlight potentially important genotype–environmental factor correlations.

## Introduction

The innate immune sensing apparatus, as originally proposed by Janeway (1), serves two important functions: first, it is a means through which the host can recognize and respond rapidly to microbial pathogens; second, the innate immune machinery directs and regulates the ensuing adaptive immune responses so as to achieve maximum efficiency against the invading pathogens. Over the past 25 years, extensive experimental evidence has been amassed in support of both of these essential functions (2). Particularly, recognition of conserved structures of pathogens by pattern recognition receptors such as toll-like receptors (TLRs) have been demonstrated to orchestrate both innate and adaptive immune responses (3, 4). Upon binding to pathogen-associated molecular patterns, most TLRs signal through myeloid differentiation primary response 88 (MyD88), an essential cytoplasmic adaptor protein that links triggering of TLRs and IL-1/IL-18 receptors to downstream activation of IL-1 receptor-associated kinases (IRAKs) and the nuclear factor-kappa B (NF-κB) (5). In turn, this initiates the production of various pro-inflammatory and immunoregulatory cytokines that control the subsequent development of antimicrobial B and T cell responses (6, 7). However, the requirement of TLR/MyD88 pathway for antibody responses has been challenged by several studies demonstrating T-cell-dependent antibody synthesis in the absence of TLR/MyD88 signaling (8).

The importance of the TLR-MyD88 pathway in host defense against a variety of microbial pathogens has been extensively demonstrated (9–20). Despite this critical requirement for TLR/MyD88 pathway for host defense against microbial infections, other studies demonstrated that it is dispensable for the generation of antibody responses, particularly in response to experimental infections by *Salmonella enterica* serovar Typhimurium (hereafter referred to as *S. typhimurium*) (17, 21, 22) and *Borrelia burgdorferi* (23). These apparently conflicting findings even extend to the requirement of MyD88/TLR pathway for protective adaptive immunity to *Salmonella* infection. While one study reported that MyD88 deficiency had little effect on protection (22), we and others have shown that MyD88-deficient mice are profoundly susceptible to infection (17, 21). *Salmonella typhimurium* bacteria are Gram-negative, food and water-borne pathogens that cause annually millions of cases of acute gastroenteritis, fever, and septicemia, representing a significant public-health problem worldwide (24, 25). Notably, damage of host tissues during *Salmonella* infections has provided a link between *Salmonella* outbreaks and the development of autoimmune diseases (26–28). However, the mechanisms behind these phenomena remain poorly understood, necessitating a better understanding of the host protective mechanisms in *Salmonella* infections.

To gain further insight into the role of TLR-MyD88 signaling in the immune response against *Salmonella*, we previously demonstrated that the inherent susceptibility of MyD88-deficient (MyD88^−/−^) mice to *Salmonella* infection is linked to a defective production of inflammatory cytokines and impaired recruitment of immune cells to the infection site (17). Despite the observed defects, MyD88^−/−^ mice produced increased levels of anti-*Salmonella* IgG antibodies (17), suggesting that *Salmonella* dysregulates the adaptive immune response. Here, we report a follow-up of our previous findings in which we characterized the activation state of innate (myeloid cells) and adaptive (T and B lymphocytes) immune responses from MyD88^−/−^ mice in response to an attenuated strain of *S. typhimurium*, designated BRD509E (29, 30). Furthermore, we determined the underlying mechanism of the hypergammaglobulinemia response in BRD509E-infected MyD88^−/−^ mice as well as its implications for autoreactive B cell responses.

## Materials and Methods

### Bacterial Strains

For the current studies, we have used an attenuated *aroA*^−^*/aroD*^−^ double auxotrophic mutant strain of *S. typhimurium* (BRD509E) cultured and prepared as previously described (30, 31). Where indicated, we also utilized a strain of *Acinetobacter baumanii* (designated NM97), a clinical isolate from Tawam hospital, which was kindly provided by Dr. Tibor Pal (Department of Medical Microbiology and Immunology, College of Medicine and Health Sciences, United Arab Emirates University). Heat-killed (HK) *Salmonella* was prepared by incubating log-phase bacterial suspension at 65°C for 1 h.

### Mice

C57BL/6 wild-type mice (MyD88^+/+^) were purchased from the Jackson Laboratory (Bar Harbor, ME, USA). MyD88-deficient mice (MyD88^−/−^) were generously provided by Dr. Shizuo Akira (Osaka University, Japan) (32) through Dr. Richard Flavell (Yale University School of Medicine, USA). Mice were bred in our animal facility and maintained in filter-topped isolator cages on Bactrim-supplemented water. Mice were taken off antibiotic for at least 7–10 days before use in any experiment. All animals were routinely used at 8–12 weeks of age when the bacteria were inoculated intraperitoneally (i.p.). All studies involving animals were conducted in accordance with and after approval of the animal research ethics committee of the College of Medicine and Health Sciences, United Arab Emirates University. In some experiments, sera from 8-week-old female autoimmune MRL/MpJ-Fas^lpr^ (MRL-lpr) mice (Jackson Laboratory) were used for comparative determination of anti-double-stranded DNA (dsDNA) titers.

### Enumeration of Bacteria in Target Organs and Fecal Pellets

Procedures for determination of bacterial loads have been detailed elsewhere (17, 33). Fecal CFUs were determined at the indicated time points by streaking fecal suspension on *Salmonella*–*Shigella* agar plates containing ampicillin and streptomycin. Similarly, bacterial CFUs were also determined in spleen and liver homogenates prepared in cold sterile saline.

### Spleen Cell Preparation and Enrichment

Erythrocyte-depleted spleen single cell suspensions were prepared as described previously (31). Purification of CD4^+^ T cells and CD11b^+^ myeloid subpopulations was done using magnetic bead separation on an autoMACS cell sorter (Miltenyi Biotec, Germany) according to manufacturer’s instructions. The purity of CD4^+^ T cells was always between 90 and 95% and myeloid cells between 80 and 85%.

### Phenotyping of Splenic Immune Cell Subsets

Processing of spleen cells for flow cytometric analysis was carried out as detailed previously (34). Immunophenotyping of splenic myeloid cells was done using the following panel of conjugated mAbs: CD11b-eFluor780, Gr-1-FITC, Sca-1-PE, and CD80-APC (all from Biolegend or eBioscience, San Diego, CA, USA). For the analysis of splenic T population, we used a panel of mAbs consisting of CD3-FITC, CD4-PE-Cy7, CD8α-APC-Cy7, CD25-APC, and Sca-1-PE. Splenic B lymphocytes were analyzed using the following mAb panel: CD19-PE-Cy7, Sca-1-PE, CD80-APC, and CD86-FITC. In all staining groups, 7-AAD dye was included in order to exclude non-viable cells from the analysis. Data were collected on 30,000 cells using BD FACSCantoII cytometer and analyzed using BD FACSDiva software.

### Analysis of Intracellular Cytokine Levels

The levels of intracellular cytokines were assessed as recently described (35), with minor modifications. Briefly, spleen cell suspensions were seeded in 24-well plates and remained unstimulated or were stimulated with anti-CD3 (clone 2C11) mAb (5 μg/well) plus 100 µl of anti-CD28 (clone 37.N.51) at 20 μg/well for 24 h at 37°C in the presence of brefeldin A for the last 4 h of culture (GolgiPlug; BDcytofix/cytoperm plusTM solution kit, BD Biosciences). After overnight incubation, cells were harvested, stained with CD4-APC-Cy7 mAb (Biolegend), and intracellularly (BD Cytofix/Cytoperm Plus #555028) with anti-IFNγ-PE-Cy7 plus anti-IL4-APC or anti-IFNγ-PE-Cy7 plus anti-IL10-PE or isotype controls (all from Biolegend). Cells were read on a BD FACSCanto II and the data analyzed using BD FACSDiva software.

### Cytokine Release

Purified splenic CD4^+^ T cells were cultured in the presence or absence of plate-bound mAbs specific to murine CD3 and CD28 molecules, as described above. After overnight incubation, supernatants were harvested and analyzed for IFNγ, IL-4, and IL-10 cytokines by ELISA following the manufacturer’s instructions (BD Biosciences).

### Gene Expression Analysis

Expression of target gene from purified and CD11b^+^ myeloid and CD4^+^ T cells was evaluated using qRT-PCR as previously described (34) using TaqMan reverse transcription reagents (Applied Biosystems, Foster City, CA, USA) and following the manufacturer’s protocol. Briefly, TaqMan primers and probes (all from Applied Biosystems) were used to study the expression of various cytokines (including BAFF, IL-4, IL-6, IL-10, IL-12p35, IL-17F, IL-21, IFN-γ, and TGF-β), transcription factors (T-bet, GATA-3, RoRγ, Bcl-6, Foxp3), inducible nitric oxide synthase (iNOS), and the checkpoint protein PD-1. The levels of transcripts were normalized according to the ΔCq method to respective mRNA levels of the housekeeping gene hypoxanthine guanine phosphoribosyltransferase (HPRT).

### Measurement of Antibacterial Antibodies

Serum samples were obtained at the indicated time points post i.p. infection with 200 CFUs/mouse (for live *Salmonella*), 2 × 10^5^ CFUs/mouse (for HK *Salmonella*), or 1 × 10^5^ CFUs/mouse of *A. baumanii*. The presence of *Salmonella*-specific or *Acinetobacter-*specific antibodies of IgG1, IgG2c, IgG3, and IgM isotypes were determined by ELISA as previously described (17, 31). Maxisorb microplates (Nunc, Roskilde, Denmark) were coated with 1 × 10^6^/ml HK BRD509E overnight. After blocking (1% BSA, 5% sucrose, 0.05% NaN_3_ in 1XPBS, pH 7.2–7.4), serum samples were titrated serially in the reagent diluent (1% BSA in 1XPBS, pH: 7.2–7.4) and incubated at room temperature (RT) for 2 h. After washing, the plates were incubated for 2 h with the respective biotin-conjugated anti-mouse Ig isotypes (goat anti-IgM, Jackson Immunoresearch, West Grove, PA, USA; goat anti-IgG1, goat anti-IgG2c, and rat anti-IgG3, all from Southern Biotech, Birmingham, AL, USA) followed by Streptavidin-HRP. The plates were developed using TMB substrate and read at 450 nm using a TECAN microplate reader (Maennedorf, Switzerland). For the quantitative determination of anti-*Salmonella* Ab concentrations, wells were coated with 0.1 µg/ml of goat anti-mouse Ig (H + L) Ab (Southern Biotech). After blocking with 2% BSA/PBS (pH 7.2) buffer, the standard curve was generated using specific mouse isotype Abs (IgG1 or IgG2c; starting concentration 50 ng/ml). For the rest of the plate, serum samples from individual animals were serially titrated in duplicate. The test plate was then incubated at RT for 2 h, washed extensively, and received appropriate secondary Abs (biotin-conjugated, isotype-specific Abs) and incubated for an additional 2 h at RT. After another series of washings, streptavidin-HRP was added and the plates developed using TMB, as described above.

### Determination of Serum Autoantibodies

The levels of autoantibodies directed against dsDNA, thyroglobulin, and mouse IgG rheumatoid factor (RF) were assessed by ELISA following a published method with modifications (36). For the detection of dsDNA-specific Abs, Maxisorp plates were coated with 1 µg/ml of dsDNA (from calf thymus; Sigma) diluted in borate-buffered saline (BBS) overnight at 4°C. After three wash cycles, wells were blocked for 1 h at RT with BBS supplemented with 0.4% Tween 20 and 1.0% BSA. Serum samples (1/500 to 1/1,000 final dilutions) were then added and incubated for 2 h. After washing with BBS, the plates were incubated with biotin-conjugated goat-anti-mouse IgG, Fcγ-specific Ab (Jackson Immunoresearch) for 2 h at RT. Finally, the plates were incubated with Streptavidin-HRP and developed with TMB, as described above. For the detection of RF and anti-thyroglobulin antibodies, Maxisorp plates were coated overnight at 4°C with 1 µg/ml of either mouse IgG (Jackson Immunoresearch) for RF assay or thyroglobulin (from bovine thyroid; Sigma) diluted in 0.05 M carbonate buffer (pH = 9.6). Following three washes with PBS containing 0.05% Tween, the wells were blocked with PBS/Tween/1% BSA for 2 h at RT. Serum samples, diluted to 1/1,000, were added in duplicate and incubated for 2 h at RT. The plates were then washed and developed using the same procedure described above.

### Detection of Antinuclear Antibodies (ANA) by Immunofluorescence Using HEp-2 Cells

Serum ANA were detected by indirect immunofluorescence using a fluorescence-based HEp-2 ANA kit, according to the manufacturer’s protocol (INOVA Diagnostics, San Diego, CA, USA). Diluted serum samples (1/80) and controls were incubated for 30 min at RT in a moist chamber. After gently rinsing with PBS, FITC-conjugated goat anti-mouse IgG (Pierce,) was added and incubated for 30 min, following which the mounting media was added and coverslip placed. The fluorescence was visualized at 40× magnification using an Olympus BX51 fluorescent microscope (Olympus Corporation, Japan).

### Histopathological Kidney Analysis

Kidney biopsies were fixed in 4% paraformaldehyde, embedded in paraffin, and assayed for immune complex deposition. Tissue sections were stained with biotin-goat anti-mouse IgG (Fcγ-specific; 1/2,000 dilution) for 2 h followed by streptavidin-HRP (BD Biosciences) for 1 h. The peroxidase activity was determined using DAB chromogen (BD Biosciences). After washing, hematoxylin was added and sections were then rehydrated, mounted with DPX, and viewed under 20× and 40× light microscope.

### Statistical Analysis

Statistical significance was analyzed using Student’s *t*-test, using the statistical program of GraphPad Prism software (San Diego, CA, USA). Differences between experimental groups were considered significant when *p* values were <0.05.

## Results

### Persistent Infection by Attenuated *Salmonella* Strain in MyD88^−/−^ Mice

**Figure 1:**
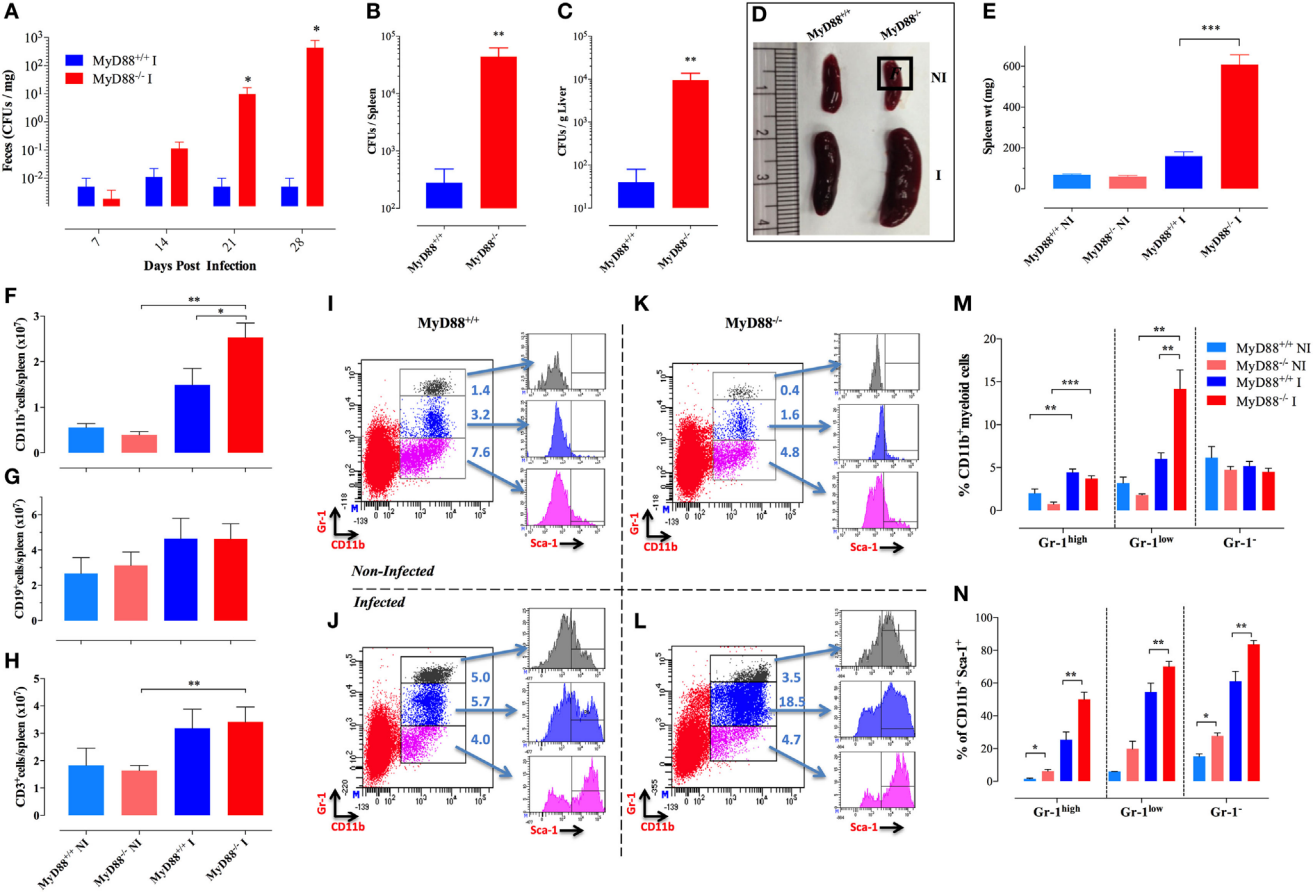
Splenomegaly and inflammatory myeloid cell influx in *Salmonella*-infected MyD88^−/−^ mice. **(A–C)** WT (MyD88^+/+^) and MyD88^−/−^ mice were infected i.p. with BRD509 (200 CFUs/mouse). Bacterial shedding in the feces was followed for 4 weeks **(A)**. Bacterial loads in spleen **(B)** and liver **(C)** at day 28 postinfection are shown. **(D,E)** Mice were infected with BRD509 (3,000 CFUs/mouse) and sacrificed at day 21. **(D)** Spleens of non-infected (NI) and infected (I) groups are shown. **(E)** Spleen weights of the different experimental groups. Each data point represents the mean ± SEM of 6–10 mice per group. **(F–H)** Absolute cell counts of CD11b^+^ myeloid cells **(F)**, CD19^+^ B lymphocytes **(G)** and CD3^+^ T lymphocytes **(H)** per spleen. **(I–N)** Flow cytometric analysis of splenic myeloid cells and activation status. Splenocytes of NI **(I,K)** or *Salmonella*-infected **(J,L)** MyD88^+/+^ **(I,J)** or MyD88^−/−^ **(K,L)** mice were analyzed for CD11b and Gr-1 positivity. Based on Gr-1 expression levels, three distinct CD11b^+^ populations could be discerned, CD11b^+^Gr-1^high^ (top panel), CD11b^+^Gr-1^low^ (middle panel), and CD11b^+^Gr-1^−^ (bottom panel). Each of these subpopulations was further analyzed for the expression of Sca-1 activation marker. Results of individual mice are shown. **(M)** Percentage of CD11b^+^ cells expressing high, low, or negative levels of Gr-1 protein in NI or BRD509-infected MyD88^+/+^ or MyD88^−/−^ mice. **(N)** Alterations in expression of Sca-1 protein in the different myeloid subpopulations. The results are representative of at least three to four independent experiments. Asterisks denote statistically significant differences between MyD88^−/−^ and MyD88^+/+^ mice (*p < 0.05; **p < 0.01; ***p < 0.001).

We have previously demonstrated that MyD88^−/−^ mice are susceptible to infection by an attenuated strain of *S. typhimurium* (BRD509E) (17, 30). While the lethal dose 50 (LD_50_) of the BRD509E strain in C57BL/6 mice, when given i.p., is ~5 × 10^6^ CFUs per mouse, the equivalent LD_50_ in MyD88^−/−^ mice is ~400 CFUs/mouse, more than 10,000-fold lower than that of C57BL/6 mice. In this context, while i.p. inoculation of BRD509E (at doses as low as 4 × 10^2^ CFUs/mouse) resulted in high mortality rates in MyD88^−/−^ mice, its oral administration led to the establishment of a chronic infection that lasted for more than 6 months (17). Here, we examined further the extent of the susceptibility to BRD509E in MyD88^−/−^ mice. We first followed the bacterial shedding in the feces over a 4-week period after the animals were infected i.p. with a low dose of BRD509E (200 CFUs/mouse). In wild-type MyD88^+/+^ mice, only a low level of fecal shedding of *S. typhimurium* organisms (~10 CFUs/g feces) was observed 2 weeks postinfection (Figure 1A). By day 21, as the infection was phenotypically resolved, a minimal fecal shedding of *S. typhimurium* organisms was observed. In contrast, MyD88^−/−^ mice exhibited a persistent, progressively worsening infection accompanied by increasing levels of bacterial shedding over the 4-week observation period, reaching >10^5^ CFUs/g feces (Figure 1A). This observation correlated with significantly elevated bacterial burden in the spleen and liver of infected MyD88^−/−^ mice, where the bacteria recovered was 158- and 240-fold greater, respectively, than in infected MyD88^+/+^ mice (Figures 1B,C). Thus, MyD88^−/−^ mice fail to control the replication of BRD509E strain irrespective of the challenge dose.

### Increased Counts and Activation of Splenic Myeloid Cells Following *Salmonella* Infection in MyD88^−/−^ Mice

To gain novel insights into the role of MyD88 in the immune response against *Salmonella*, MyD88^−/−^ mice were infected with ~3 × 10^3^ CFUs/mouse of BRD509E strain, which corresponds to an intermediate dose when compared to our previous report (17), and changes in spleen cellularity were analyzed at day 21 postinfection. MyD88^−/−^ mice exhibited a protracted state of splenomegaly, ~3.8-fold larger than in wild-type counterparts (Figures 1D,E). Analysis of splenic immune cells demonstrated that MyD88^−/−^ mice displayed a significant increase in the number of CD11b^+^ myeloid cells in comparison to infected MyD88^+/+^ counterparts (Figure 1F). Meanwhile, no change was observed in absolute counts of T and B lymphocytes (Figures 1G,H).

To characterize the different subpopulations of CD11b^+^ myeloid cells in the spleen of infected animals, we performed flow cytometric analysis using a combination of mAbs to CD11b and Gr-1 proteins as well as the activation markers Sca-1, CD80, and MHC class II. Based on staining with mAbs to CD11b and Gr-1, three CD11b^+^ myeloid subpopulations (Gr-1^high^, Gr-1^low^, and Gr-1^−^) could be discerned (Figures 1I–L). The Gr-1^high^ cells represent granulocytes and are negative for MHC class II and CD80 expression (data not shown). The CD11b^+^Gr-1^low^ and Gr-1^−^ cells represent monocyte lineage cells and are positive for MHC class II (data not shown). By day 21 postinfection, the levels of splenic Gr-1^high^, and Gr-1^low^ subpopulations were still elevated in both infected MyD88^−/−^ and MyD88^+/+^ mice as compared to non-infected (NI) counterparts (Figures 1J,L). Splenic Gr-1^low^ monocytes were the predominant cell type in infected MyD88^−/−^ mice (Figure 1M), accounting for the observed protracted splenomegaly (Figure 1D). Sca-1 is an IFNγ-inducible activation marker on myeloid and lymphoid cells (37) and it has been shown to be upregulated in *Salmonella* infections (29). Analysis of Sca-1 expression on splenic myeloid cells confirmed its upregulation on all three subpopulations (Figures 1I–L,N) with the extent of activation being consistently higher in infected MyD88^−/−^ mice. This is most likely a reflection of the chronicity of *Salmonella* infection in these mice.

**Figure 2:**
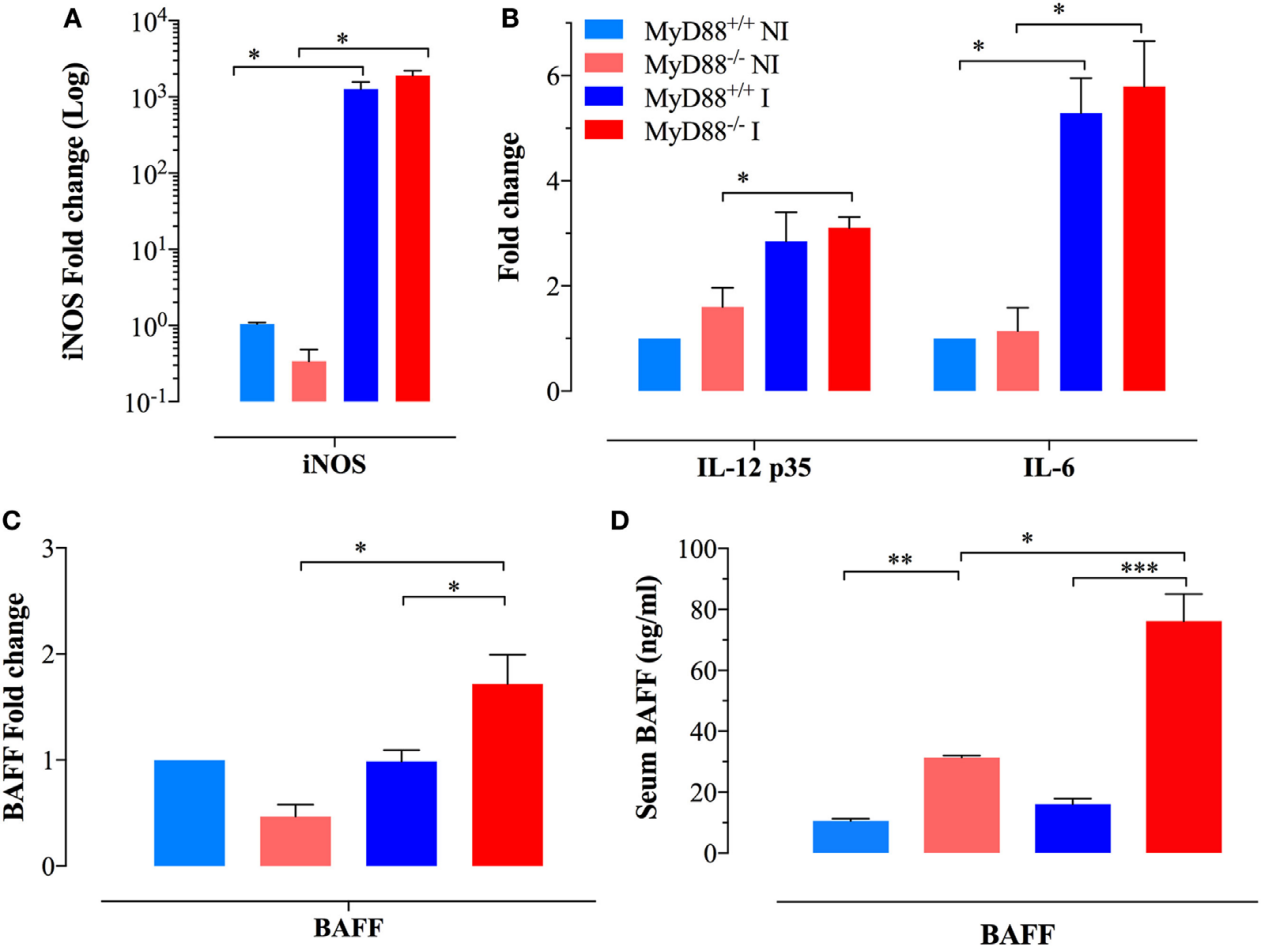
Analysis of cytokine gene expression and serum levels in myeloid cells. Gene expression levels of **(A)** inducible nitric oxide synthase (iNOS) and **(B)** IL-12p35 and IL-6 in CD11b^+^ splenic myeloid cells purified from the different experimental groups. Each data point represents the mean ± SEM of three to four mice per group. **(C)** Analysis of BAFF gene expression in purified myeloid cells [*n* = 3 for non-infected (NI) groups and *n* = 8 for infected groups]. Alterations in gene expression are depicted as fold-change compared to NI WT mice. **(D)** Analysis of systemic BAFF levels in mouse sera by ELISA. Each data point represents the mean ± SEM of three (NI) or eight (infected) mice per group. Analysis was done on whole sera or purified myeloid cells obtained from NI or *Salmonella*-infected mice at day 21 postinfection. Data are compiled from two independent experiments (*p < 0.05; **p < 0.01; ***p < 0.001).

Given the persistent infection and increased infiltration of inflammatory myeloid cells into the spleens of MyD88^−/−^ mice at day 21 of infection, CD11b^+^ cells were purified from different experimental groups and assessed for the expression of inflammatory mediators. We observed an elevated iNOS transcriptional activity in myeloid cells which reached >1,000-fold higher levels in both MyD88^+/+^ and MyD88^−/−^ myeloid cells compared to NI animals (Figure 2A), indicative of an ongoing pro-inflammatory milieu in both mouse strains. In agreement, equivalent levels of IL-12p35 and IL-6 were also evident in both NI and infected MyD88^+/+^ and MyD88^−/−^ mice (Figure 2B). Of note, a significantly enhanced BAFF expression level was observed in infected MyD88^−/−^ mice compared to wild-type mice (Figure 2C). Given the important role of BAFF in driving autoreactive B cell responses (38, 39), we also determined the levels of BAFF cytokine in mouse sera. Measurement of systemic BAFF levels revealed a 4.9-fold increase in BAFF in infected MyD88^−/−^ mice compared to infected MyD88^+/+^ animals (Figure 2D). Interestingly, this analysis also showed significantly higher (threefold) BAFF serum levels in uninfected MyD88^−/−^ mice compared to their wild-type counterpart (Figure 2D). Additionally, *Salmonella* infection induced significant elevation (2.5-fold) in serum BAFF levels in MyD88^−/−^ mice compared to uninfected counterpart (Figure 2D). Collectively, these findings suggest a hyperinflammatory state of myeloid cells from MyD88^−/−^ mice in the context of a chronic infection with attenuated *Salmonella*. This is also combined with significantly elevated serum BAFF levels in MyD88^−/−^ mice.

### Enhanced T Lymphocyte Activation in the Spleens of *Salmonella*-Infected MyD88^−/−^ Mice

**Figure 3:**
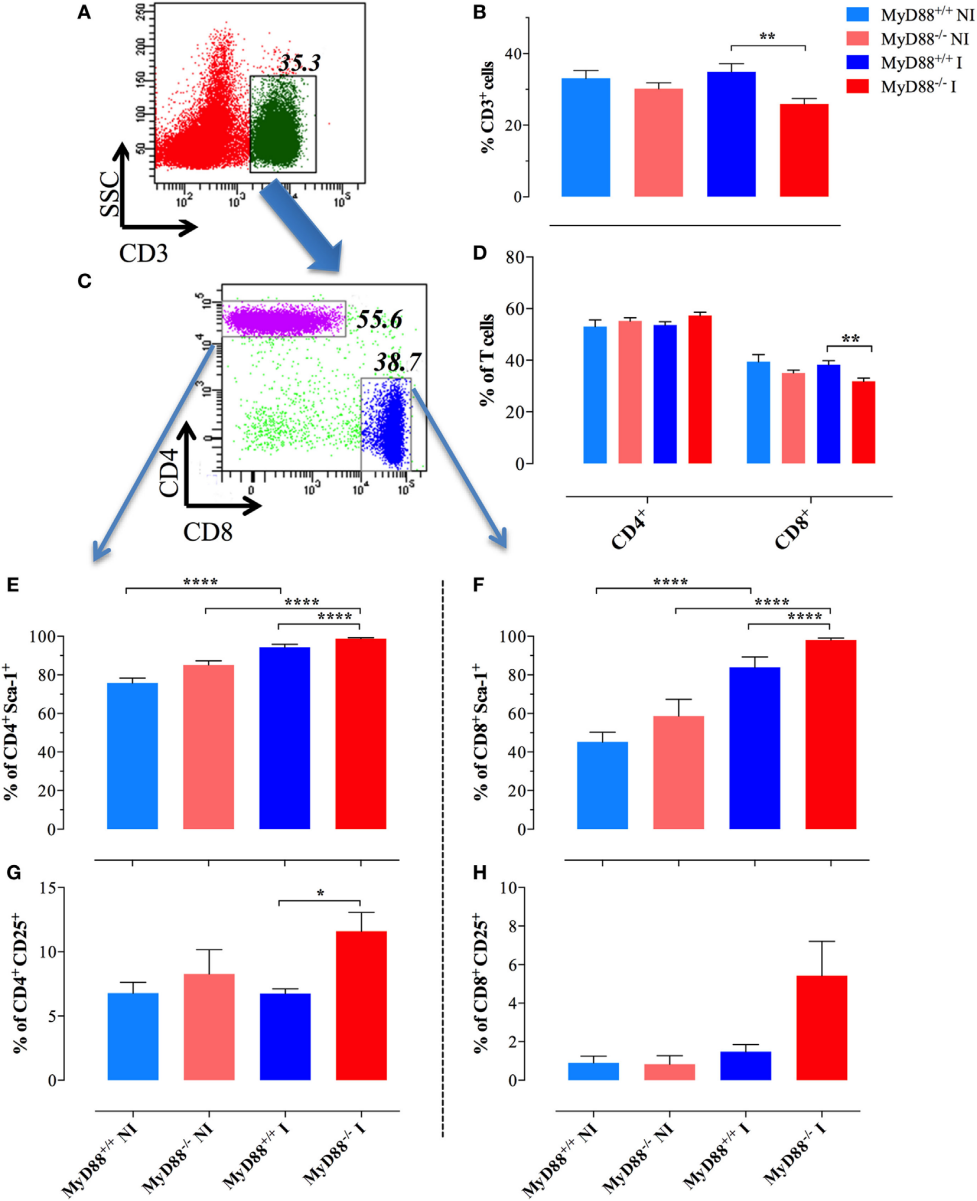
Changes in splenic T lymphocyte populations following infection. A representative dot plot of non-infected (NI) WT splenocytes showing gated CD3^+^ T lymphocytes **(A)** and CD4 and CD8 subpopulations **(C)** within gated CD3^+^ cells. **(B)** Changes in the percent of CD3^+^ lymphoid cells in the whole spleen. **(D)** Changes in the ratios of CD4^+^ and CD8^+^ cells within the T cell populations. Analysis was done on day 21 postinfection with BRD509 (*n* = 4–6 for NI and 8–10 for infected groups). Changes in the percent of CD4^+^ **(E,G)** and CD8^+^ **(F,H)** T cells positive for Sca-1 **(E,F)** or CD25 **(G,H)** are shown (*p < 0.05, **p < 0.01, ***p < 0.001). The results are representative of three independent experiments.

Considering the interconnection between innate and adaptive immune responses (3, 40), the abnormal activation displayed by splenic myeloid cells of MyD88^−/−^ animals suggested the possibility that *Salmonella* infection could also affect the activation of adaptive immune cells. The frequency of CD3^+^ T cells in NI wild-type mice was 33.1 ± 2.1 and was essentially unchanged (34.9 ± 2.2) at day 21 postinfection (Figures 3A–C). Despite no changes in the absolute lymphocyte counts when comparing infected MyD88^+/+^ with MyD88^−/−^ mice (Figure 1H), the latter showed a slightly lower frequency of CD3^+^ cells (25.9 ± 1.5) in the T lymphocyte compartment (Figure 3B). Since the frequency of CD4^+^ T cells was not abnormal in MyD88^−/−^ mice, the reduced CD3^+^ cells in the T lymphocyte compartment was probably due to low percentage of CD8^+^ T cells (Figure 3D). Most important, phenotypic analysis of T cells upon *Salmonella* infection demonstrated an increased expression of Sca-1 on CD4^+^ and CD8^+^ T cells from both mice strains (Figures 3E,F; Figure S1 in Supplementary Material). However, the upregulation of Sca-1 expression was significantly higher on T cells from MyD88^−/−^ mice compared to wild-type mice. Furthermore, while we did not detect increased CD25 expression on T cells from infected wild-type mice when compared to NI animals, the absence of MyD88 resulted in a strong upregulation of this activation marker (Figures 3G,H; Figure S1 in Supplementary Material). Therefore, the incapacity to control *S. typhimurium* infection in MyD88^−/−^ mice leads to a hyperactivation of innate and adaptive immune cells.

### Abnormalities in T Helper Subsets in MyD88^−/−^ Mice

**Figure 4:**
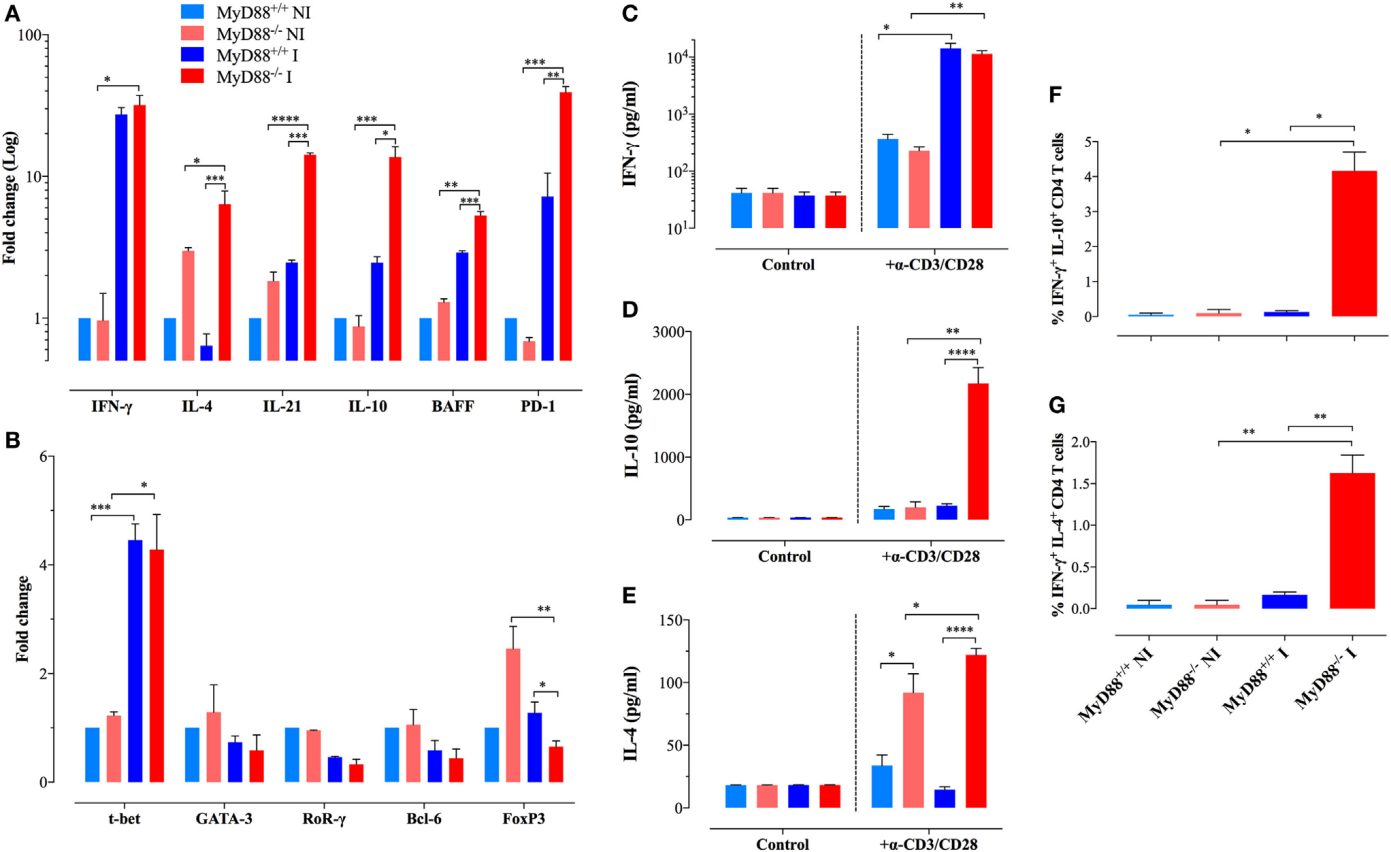
Analysis of gene expression and cytokine production profiles in purified CD4^+^ T lymphocytes. **(A,B)** Gene expression levels of key cytokines **(A)** and transcription factors **(B)** in purified CD4^+^ T cells of different experimental groups were analyzed by quantitative real-time PCR (*n* = 3–6 per group). Alterations in gene expression are depicted as fold-change compared to non-infected (NI) WT mice. Analysis was done on CD4^+^ T cells purified from NI or *Salmonella*-infected mice at day 21 postinfection. **(C–E)** Secretion of IFN-γ **(C)**, IL-10 **(D)**, and IL-4 **(E)** by purified CD4^+^ T cells after stimulation with plate-bound anti-CD3/CD28 antibodies. Data are compiled from two independent experiments (*n* = 3–6 per group). **(F,G)** Intracellular cytokine staining of purified CD4^+^ T cells. The graphs depict cumulative data showing the percent of CD4^+^ T cells co-expressing IFN-γ plus IL-10 **(F)** or IFN-γ plus IL-4 cytokines **(G)**. All determinations were done in triplicates. The data are representative of three independent experiments (*p < 0.05, **p < 0.01, ***p < 0.001, ****p < 0.0001).

We sought to further characterize the immunopathological mechanisms behind the abnormal adaptive immune cells during uncontrolled *S. typhimurium* infection. Considering the pivotal role of CD4^+^ T lymphocytes in the global control of immune responses including the host resistance against *Salmonella* (41, 42), we evaluated the gene expression in purified splenic CD4^+^ T cells of cytokines and transcription factors that specify the program of T helper cells in MyD88^+/+^ and MyD88^−/−^ mice. Transcription of IFN-γ, the main cytokine that defines the Th1 signature, was significantly elevated (27- to 32-fold) upon *Salmonella* infection in both MyD88^+/+^ and MyD88^−/−^ mice (Figure 4A). Noteworthy, CD4^+^ T cells from MyD88^−/−^ spontaneously expressed increased levels of IL-4 and IL-21 when compared to wild-type mice, which are pivotal cytokines that define Th2 (43–45) and Tfh (follicular Th cells) (46, 47) profiles, respectively. After *Salmonella* infection, the amount of IL-4 and IL-21 was strongly upregulated in CD4^+^ T cells of MyD88^−/−^, but not MyD88^+/+^, mice (Figure 4A). The expression level of IL-10, another Th2 cytokine (48), was also elevated (~14-fold) in infected MyD88^−/−^ mice (Figure 4A). Interestingly, the enhanced Tfh-specific transcriptional signature was mirrored by significant upregulation in BAFF (5.3-fold) and PD-1 (39.2-fold) gene transcription compared to control (Figure 4A). The increase in IL-4, IL-21, IL-10, BAFF, and PD-1 gene transcription was significantly higher in infected MyD88^−/−^ compared to infected MyD88^+/+^ mice. On the other hand, no significant changes were observed in the expression of IL-17 or TGF-β in CD4^+^ T cells from any mouse strain (data not shown) Overall, our data suggest that *S. typhimurium* infection exacerbates Th2 and Tfh transcriptional signatures, which are essential for the modulation of antibody production (49, 50).

In order to validate our gene expression approach, we purified CD4^+^ T cells from NI and infected mice and analyze the secretion of cytokines upon stimulation with a combination of plate-bound anti-CD3/CD28 mAbs. Both MyD88^+/+^ and MyD88^−/−^ mice secreted abundant and comparable levels of IFN-γ (Figure 4C). IL-10 secretion was only strongly detected in the supernatants of CD4^+^ T cells from infected MyD88^−/−^ mice (Figure 4D). Moreover, IL-4 secretion was detected only from MyD88^−/−^ CD4^+^ T cells, which is in agreement with the gene expression findings (Figure 4E). Interestingly, CD4^+^ T cells from uninfected MyD88^−/−^, but not MyD88^+/+^, mice could also secrete IL-4 upon induction *via* the TCR, suggesting an endogenous predisposition to Th2 responses in these mice (Figure 4E). Next, intracellular staining of cytokines in anti-CD3/CD28-activated CD4^+^ T cells was performed by flow cytometric analysis. The data revealed that while CD4^+^ T cells from infected MyD88^+/+^ mice follow the traditional Th1/Th2 polarized model, CD4^+^ T cells from MyD88^−/−^ mice co-expressed IFN-γ and IL-4 or IFN-γ and IL-10 (Figures 4F,G; Figure S2 in Supplementary Material). The rather heterogeneous cytokine profile observed in MyD88^−/−^ T cells is typical of the cytokine pattern expressed by Tfh cells (51). Recently, it was reported that naïve Th21 cells, which are implicated in autoimmune disease, do also express a combination of cytokines (IL-21 and IFNγ) typically associated with different Th cell lineages (52).

Underlying the findings described above, we observed an upregulated expression of T-bet, the transcriptional factor responsible for Th1 differentiation, in CD4^+^ T cells from both mice strains (Figure 4B). Meanwhile, NI MyD88^−/−^ mice displayed a higher spontaneous gene expression of FoxP3, the transcription factor responsible for the differentiation of T regulatory (T reg) cells, compared to wild-type mice. However, following infection, the expression of FoxP3 was downregulated to levels comparable to those from wild-type mice. No changes were observed in the expression of GATA-3, RoRγ, or Bcl-6, which are responsible for the commitment of Th2, Th17, and Tfh cells, respectively (Figure 4B). Thus, despite the skilled T lymphocytes hyperactivation profile for Th2 and Tfh signatures, suggesting unique Th cell differentiation in infected MyD88^−/−^ mice, transcription factor expression profiling in total CD4^+^ T cells showed no substantial differences between MyD88^+/+^ and MyD88^−/−^ mice, most likely due to the low sensitivity of the assay.

### MyD88^−/−^ Mice Exhibit Dysregulated Antibody Responses When Challenged With *S. typhimurium*

Apart from hyperactivation of myeloid cells including the increased expression of BAFF, a well-known factor associated with abnormal B cell responses and autoimmune diseases (53–56), we demonstrate here that MyD88^−/−^ mice develop hyperactivation of Th2 and Tfh cells. While the role of IL-4 producing cells (Th2) is still a paradox due to pro- or anti-inflammatory effects associated with the development or prevention of autoimmune diseases (57), respectively, it is clear that Tfh cells are key players for the development of humoral immunity and associated autoimmune diseases (52). Although NI and *Salmonella*-infected MyD88^+/+^ and MyD88^−/−^ mice showed similar percentages of splenic B cells (Figure S3A in Supplementary Material), B cells of MyD88^−/−^ mice exhibited higher Sca-1 expression (Figures S3B,C in Supplementary Material), suggesting hyperactivation. Analysis of costimulatory molecules on the surface of B cells demonstrated elevated levels of CD86 expression from infected MyD88^−/−^ mice when compared to MyD88^+/+^ animals (Figure S3E in Supplementary Material), while no differences were observed in the expression of CD80 molecule (Figure S3D in Supplementary Material). Thus, B lymphocytes from MyD88^−/−^ mice appear to be hyper-responsive to *Salmonella* infection.

**Figure 5:**
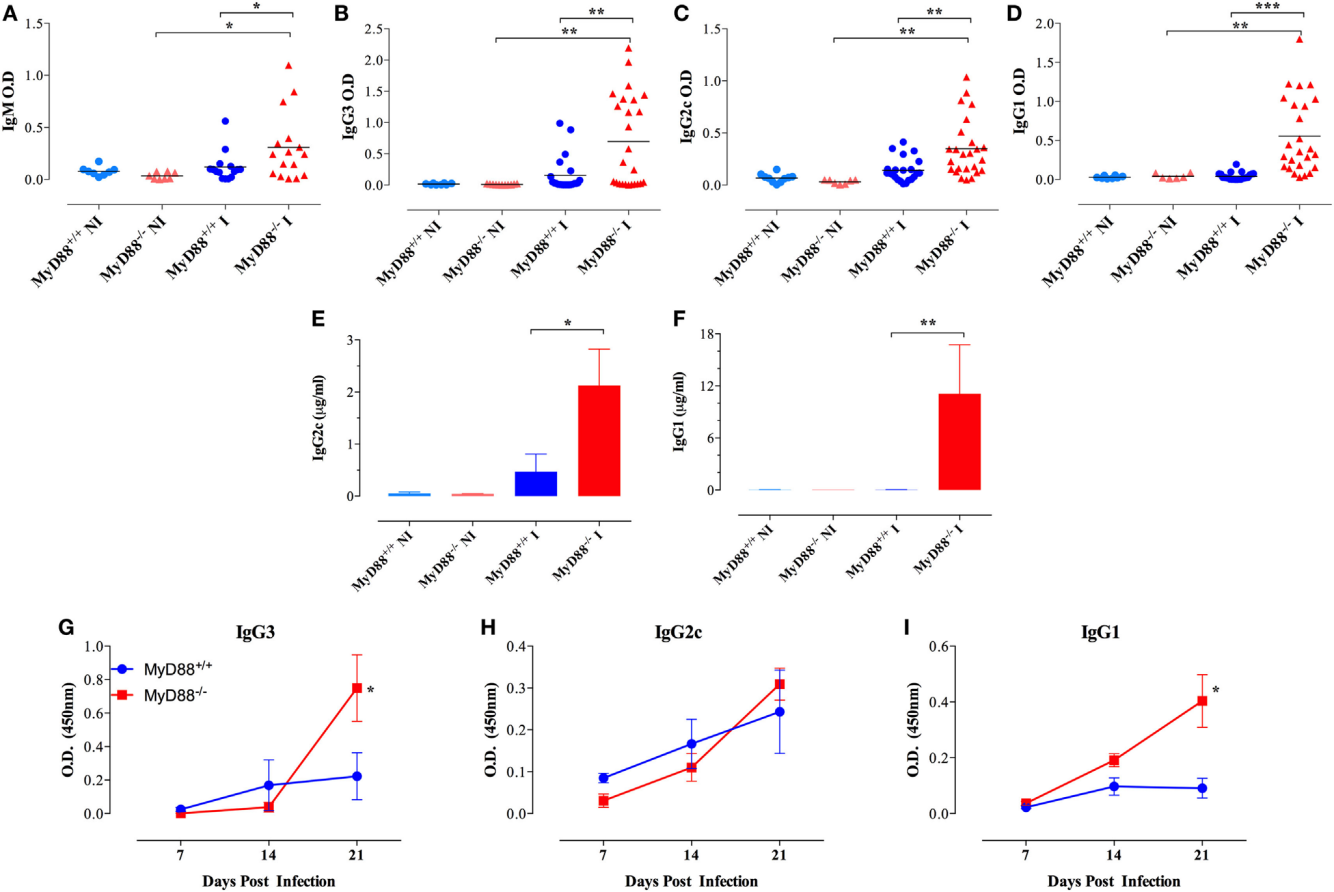
Hypergammaglobulinemia in *Salmonella*-infected MyD88^−/−^ mice. Mice were inoculated i.p. with BRD509 (~200 CFUs/mouse) and sera collected at 4 weeks and analyzed for the presence of *Salmonella*-specific IgM **(A)**, IgG3 **(B)**, IgG2c **(C)**, and IgG1 **(D)** isotypes. Quantification of *Salmonella*-specific serum IgG2c **(E)** and IgG1 **(F)** antibodies in non-infected (NI) and 3-week infected **(I)** mice. Absolute antibody levels are expressed in micrograms per milliliter serum (*n* = 3–6 per group). **(G–I)** The kinetics of development of antibodies to *Salmonella* infection was evaluated during the first 3 weeks post i.p. infection with a dose of 4,000 CFUs/mouse. *Salmonella*-specific IgG3, IgG2c, and IgG1 were determined (*n* = 3–6 per group per time point). Asterisks denote statistically significant differences between MyD88^−/−^ and MyD88^+/+^ mice (*p < 0.05; **p < 0.01; ***p < 0.001). Data are compiled from three independent experiments.

In accordance, even at a low dose of infection (equivalent to <1 LD_50_), MyD88^−/−^ mice displayed high serum levels of anti-*Salmonella* antibodies of IgM as well as IgG3 and IgG2c and IgG1 isotypes in comparison to wild-type mice (Figures 5A–D). The absolute levels of *Salmonella*-specific IgG2c and IgG1 were also measured and the data confirmed the serum titers (Figures 5E,F). The high IgG serum levels of anti-*Salmonella* antibodies in infected MyD88^−/−^ mice first became evident as early as 21 days post *Salmonella* inoculation (Figures 5G–I) and was still significantly elevated up to 2 months later, the longest period of observation (data not shown). We also measured total IgG levels and observed that in NI animals, relatively low and comparable levels of serum IgG3 and IgG2c were detected in both MyD88^+/+^ and MyD88^−/−^ mouse strains (Figures S4A,B in Supplementary Material), while IgG1 amounts in uninfected MyD88^−/−^ mice were significantly elevated compared to MyD88^+/+^ mice (2,303 ± 740 vs 626 ± 95 µg/ml, respectively; Figure S4C in Supplementary Material). In *Salmonella*-infected mice, serum IgG3 and IgG2c concentrations were significantly increased, by an average of threefold to fivefold over those in uninfected controls, in both mouse strains (Figures S4A,B in Supplementary Material), while no significant changes in serum IgG1 concentrations were observed upon infection with *Salmonella* in both mouse strains. Noteworthy, the serum levels of all three IgG subclasses in infected MyD88^−/−^ mice were higher in relation to MyD88^+/+^. Hence, in addition to showing a humoral immune response that is constitutively skewed to Th2-induced IgG isotypes, MyD88^−/−^ mice challenged with attenuated *Salmonella* display hypergammaglobulinemia due to unresolved infection.

### Presence of Self-Reactive B Cells in MyD88^−/−^ Mice Following Systemic *Salmonella* Infection

**Figure 6:**
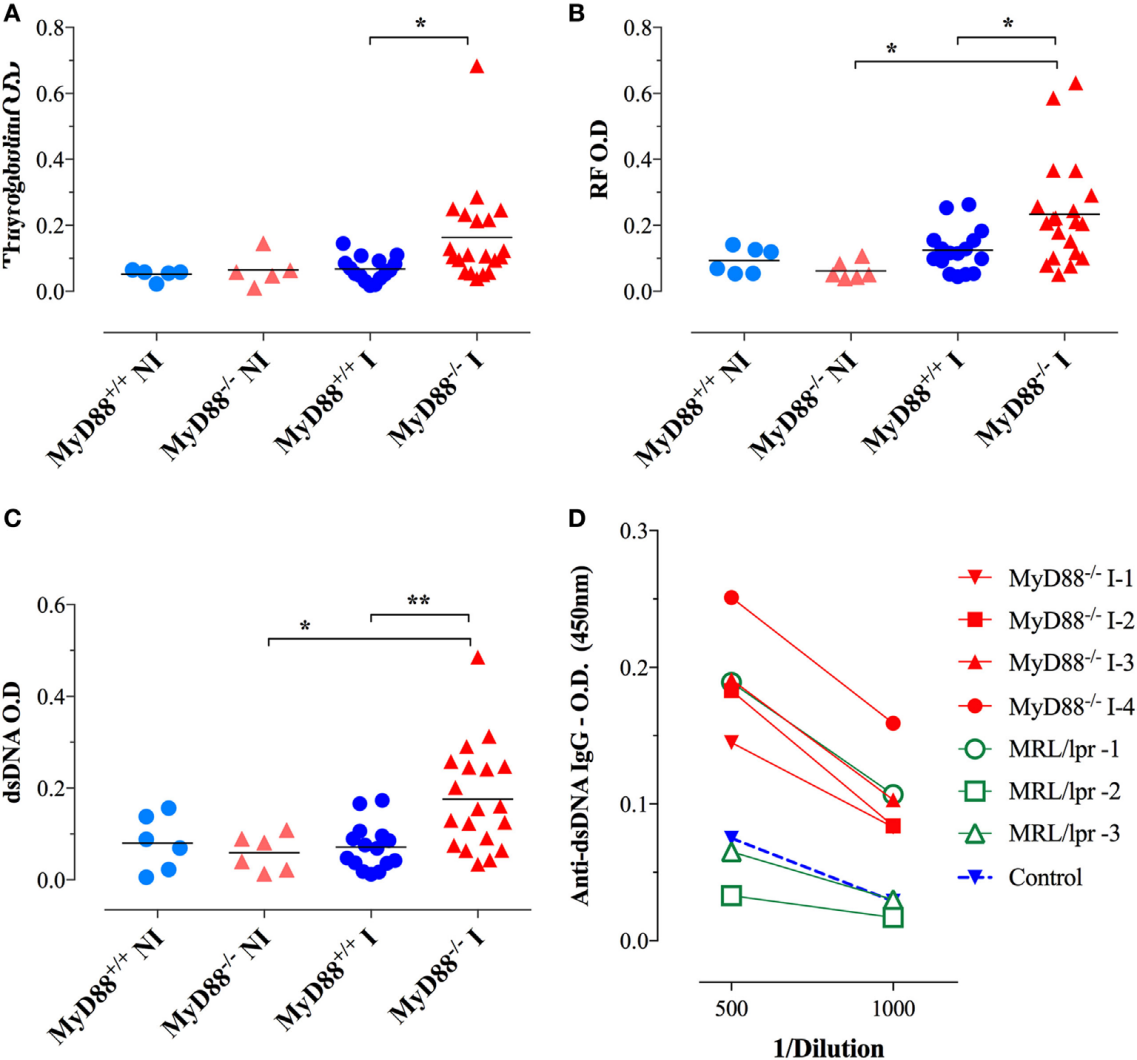
Autoantibody reactivity of MyD88^−/−^ sera after systemic *Salmonella* infection. Sera were collected 4–6 weeks following i.p. infection with BRD509 (~200 CFUs/mouse) and tested for reactivity with thyroglobulin **(A)**, rheumatoid factor [RF; **(B)**], and double-stranded DNA (dsDNA) **(C)** by specific ELISA. The cutoff for the detection of these autoantibodies was determined at 1/1,000 dilution. Data are compiled from three independent experiments. **(D)** Comparison of anti-dsDNA reactivity in sera of MRL-lpr (*n* = 3) and infected MyD88^−/−^ mice (*n* = 4). Serum reactivity with dsDNA was done at the indicated final dilutions (1/500 to 1/1,000). Non-infected sera are represented by the “Control” group. Data are representative of two independent experiments (*p < 0.05; **p < 0.01; ***p < 0.001).

Considering the immune hyperactivation and potential etiological link between *Salmonella* infections and the development of autoimmune diseases (26–28), we analyzed the generation of autoantibodies in response to BRD509E. Mouse sera were collected 4–6 weeks following i.p. infection and tested for reactivity against dsDNA, thyroglobulin, and IgG. To rule out non-specific cross-reactivity, all autoantibody ELISAs were determined at a serum dilution of 1/1,000 or greater. Moreover, since all ELISAs were run using IgG-specific secondary antibodies, all observed reactivities reflect IgG isotype autoantibodies. No significant levels of autoreactive antibodies were detectable in wild-type (MyD88^+/+^) mice before or after infection. Similarly, sera from NI MyD88^−/−^ mice had no reactivity to any of the autoantigens. On the other hand, *Salmonella*-infected MyD88^−/−^ mice displayed increased titers of autoantibodies directed against dsDNA (i.e., ANA), thyroglobulin, and IgG RF (Figures 6A–C). In order to gain further insight about the physiological significance of the observed autoantibody levels, we compared the sera of *Salmonella*-infected MyD88^−/−^ mice with that of autoimmune MRL-lpr mice for reactivity to dsDNA. The MRL-lpr serum samples were collected from 8-week-old female mice and were positive at 1/100 dilution for ANA using the Euroimmun IIFT:HEp-20-10 cell test kit (data not shown). As shown in Figure 6D, a comparison of randomly selected individual serum samples showed that the levels of dsDNA-specific IgG autoantibodies in infected MyD88^−/−^ mice were comparable to, or higher than, those of MRL-lpr mice. Taken together, the data confirm that *Salmonella* infection of MyD88^−/−^ mice induces significant levels of autoantibodies of multiple specificities.

**Figure 7:**
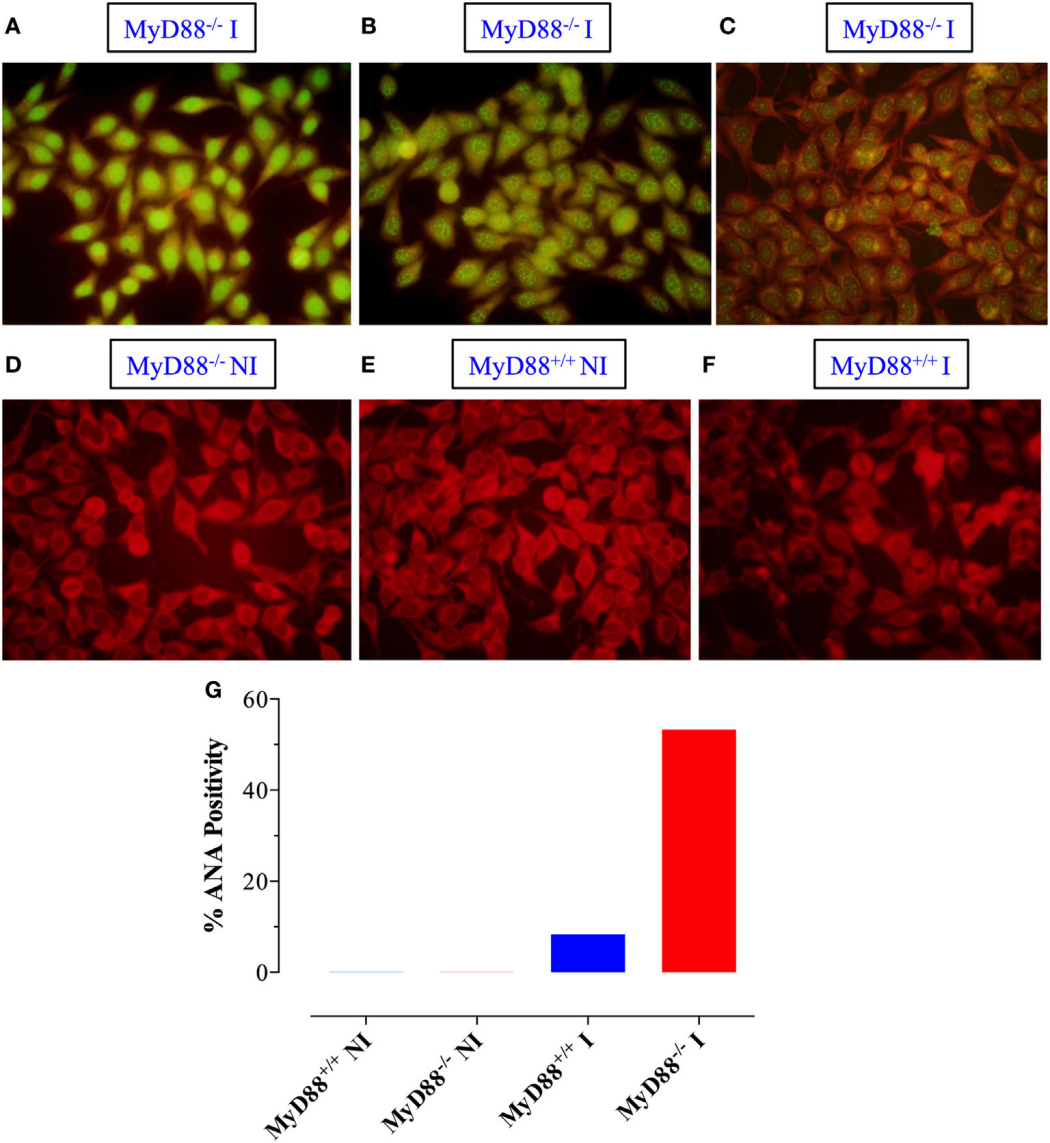
Qualitative detection of antinuclear antibodies (ANA) by immunoflurosence on HEp-2 cells. Sera from *Salmonella*-infected **(A–C)** or non-infected (NI) **(D)** MyD88^−/−^ mice were incubated with HEp-2 cells, as described in Section “Materials and Methods.” Positive staining showing homogenous **(A)**, speckled **(B)**, and cytoplasmic **(C)** patterns is shown. As a control, reactivity of sera from NI **(E)** or infected **(F)** MyD88^+/+^ mice is also shown. The percentage of sera from the different experimental groups (*n* = 5–15 mice/group) that was positive for ANA is summarized in panel **(G)**. The fluorescence was visualized at 40× magnification using an Olympus fluorescent microscope. Data are compiled from three independent experiments.

Sera were also tested for reactivity to fixed HEp-2 cells by immunofluorescence. Sera from infected MyD88^−/−^ mice exhibited different patterns of reactivity to dsDNA, ranging from homogenous to fine/coarse speckled (Figures 7A–C). Importantly, sera from uninfected mice from both groups were negative for HEp-2 staining (Figures 7D,E). While only 8% of the sera from infected MyD88^+/+^ mice (1 out of 12) were positive for HEp-2 staining (Figure 7F), approximately 53% of sera (8 out of 15) collected from infected MyD88^−/−^ mice showed strong reactivity to dsDNA in HEp-2 cells (Figure 7G). These data suggest that different nuclear antigens are being recognized by the autoantibodies produced in infected MyD88^−/−^ mice.

**Figure 8:**
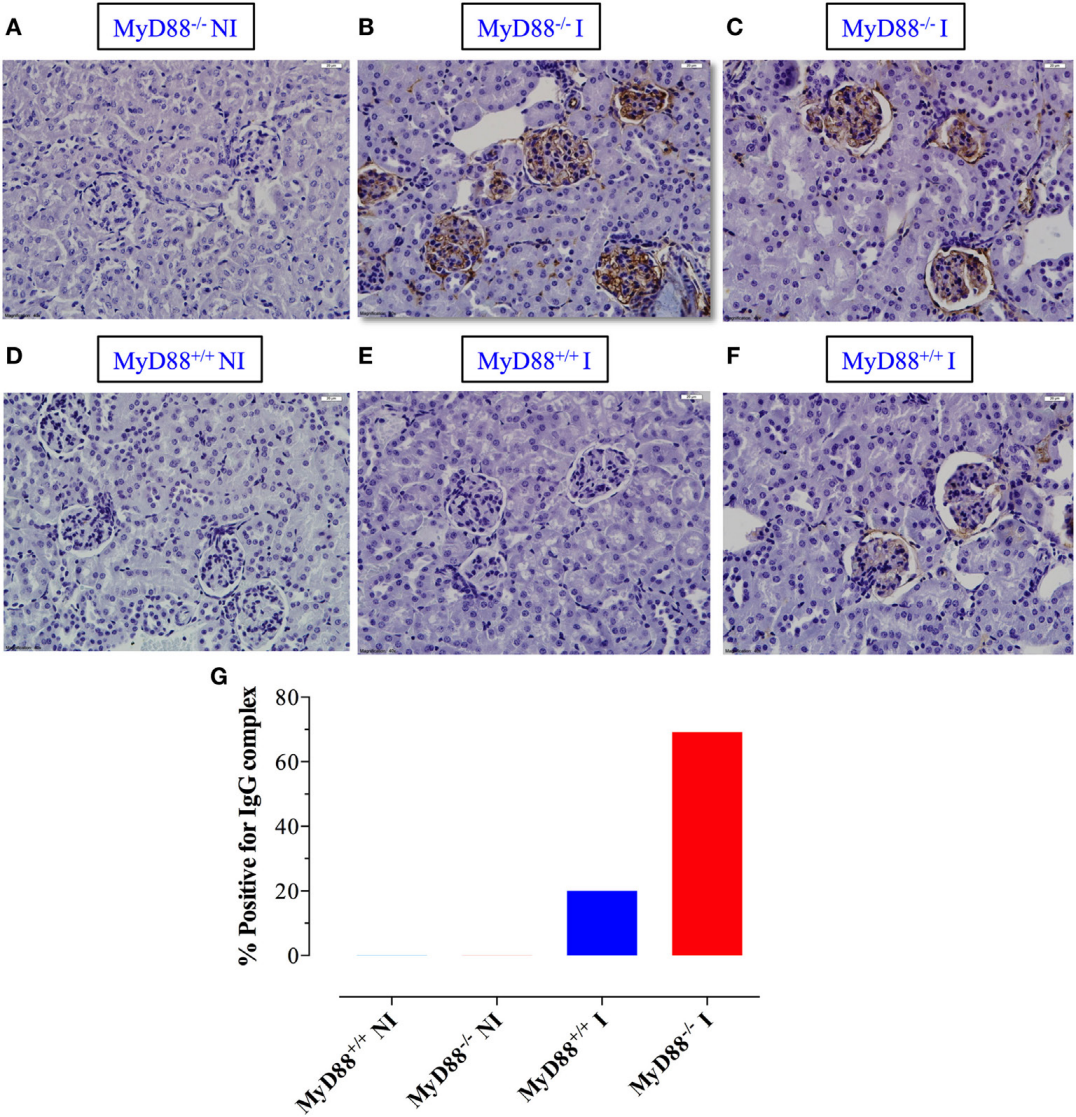
Deposition of immune complexes in *Salmonella*-infected MyD88^−/−^ mice. **(A–F)** Kidney sections were prepared from non-infected (NI) **(A,D)** and infected (I) MyD88^−/−^ **(B,C)** and MyD88^+/+^ **(E,F)** mice and stained with anti-IgG antibody, as described in Section “Materials and Methods” (*n* = 5–15 mice/group). **(G)** The percentage of mice whose kidney sections scored positive for the presence of immune deposits by DAB staining. Images were taken at 40× magnification. Data are compiled from three independent experiments.

Given the state of hypergammaglobulinemia and the increased levels of serum IgG autoantibodies in infected MyD88^−/−^ mice, we next evaluated whether this fact was associated with more abundant immune complex deposition in kidney glomeruli. No evidence of staining was detected in the glomeruli of NI MyD88^+/+^ or MyD88^−/−^ mice (Figures 8A,D). In sharp contrast, much brighter staining, indicating more deposits of immune complexes, was detected in ~69% of *Salmonella*-infected MyD88^−/−^ mice (Figures 8B,C,G) while about 20% of infected MyD88^+/+^ mice showed positive staining of glomeruli but those were generally of low level of intensity (Figures 8E,F). Taken together, our data suggest that the absence of MyD88 predisposes animals to hypergammaglobulinemia, production of autoantibodies, and deposition of immune complexes in kidney glomeruli following infection with *S. typhimurium*.

### Requirements for Infection-Induced Hypergammaglobulinemia and Autoantibody Production

**Figure 9:**
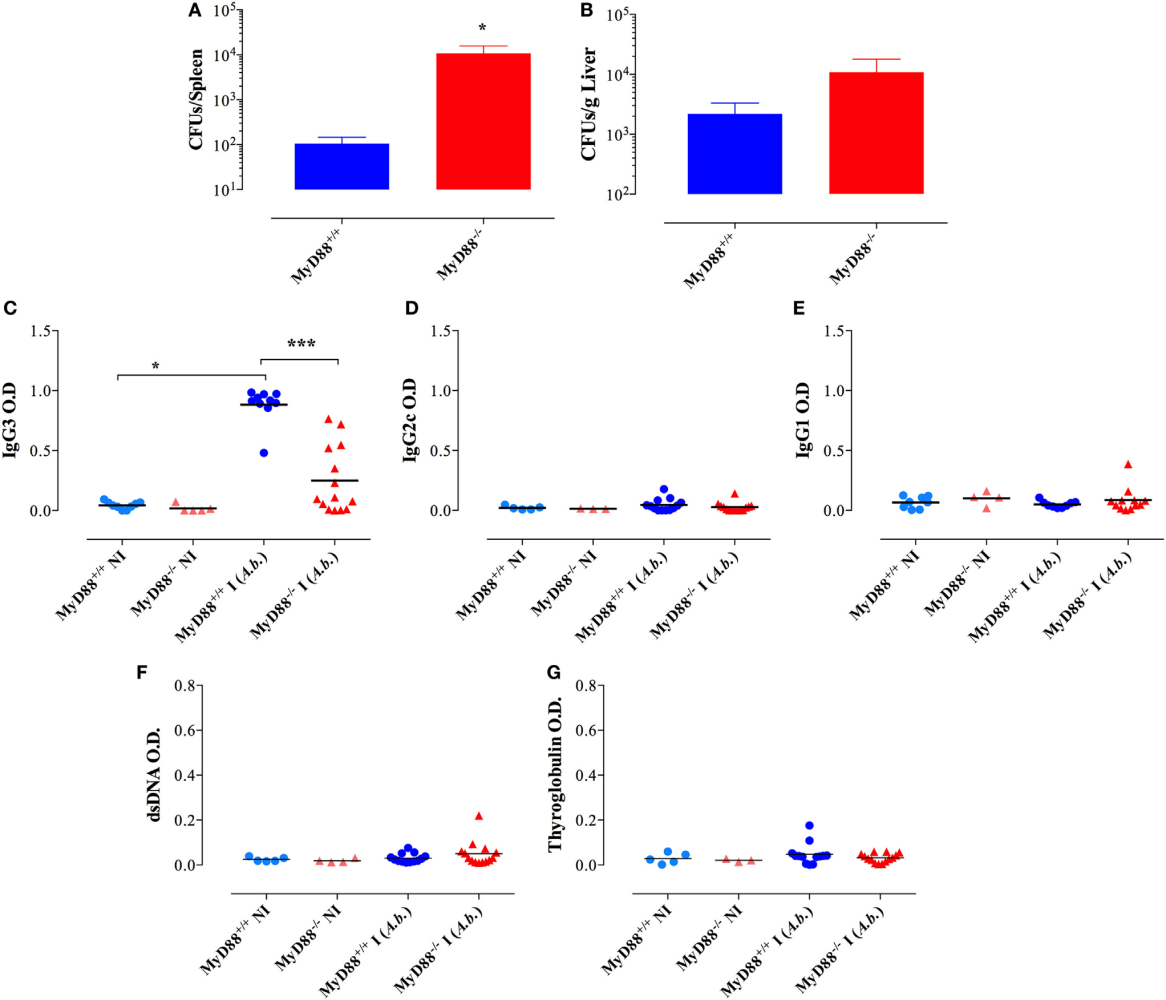
No evidence for immunoglobulin dysregulation in MyD88^−/−^ mice following infection with *Acinetobacter baumanii*. Two months post i.p. infection with a dose of ~1 × 10^5^ CFUs/mouse of *A. baumanii*, bacterial counts in spleen **(A)** and liver **(B)** were enumerated (*n* = 6–7 mice/group). Serum levels of anti-*Acinetobacter* IgG3 **(C)**, IgG2c **(D)**, and IgG1 **(E)** isotypes. **(F,G)** Serum reactivity to double-stranded DNA (dsDNA) and thyroglobulin. The cutoff for the detection of these autoantibodies was determined at 1/1,000 dilution (*p < 0.05, ****p < 0.0001). Data are compiled from two independent experiments.

The next series of experiments were designed to study the requirements for the observed hypergammaglobulinemia and autoantibody synthesis in MyD88^−/−^ mice. We first asked whether infection with another Gram-negative bacterium, *A. baumanii* (strain NM970), could lead to similarly dysregulated antibody production in MyD88^−/−^ mice. Two months following inoculation, MyD88^−/−^ mice exhibited higher bacterial loads in spleen and liver when compared to MyD88^+/+^ mice (Figures 9A,B). Infected MyD88^−/−^ mice developed mainly IgG3 antibodies specific to *Acinetobacter*, but they were significantly lower (fourfold) than those observed in infected MyD88^+/+^ mice (Figure 9C). No significant bacteria-specific IgG2c or IgG1 antibodies were detected (Figures 9D,E). In addition, *A. baumanii* did not induce antibodies directed against dsDNA and thyroglobulin (Figures 9F,G). Furthermore, there was no evidence of antibody dysregulation or autoantibody production in MyD88^−/−^ mice infected with the Gram-negative pathogen, *Escherichia coli* or with the Gram-positive bacterium Group B *Streptococcus* (*Streptococcus agalactiae*) (data not shown). Therefore, the development of autoreactive B cells in infected MyD88^−/−^ mice is dependent on the pathogen with which the animals are challenged.

**Figure 10:**
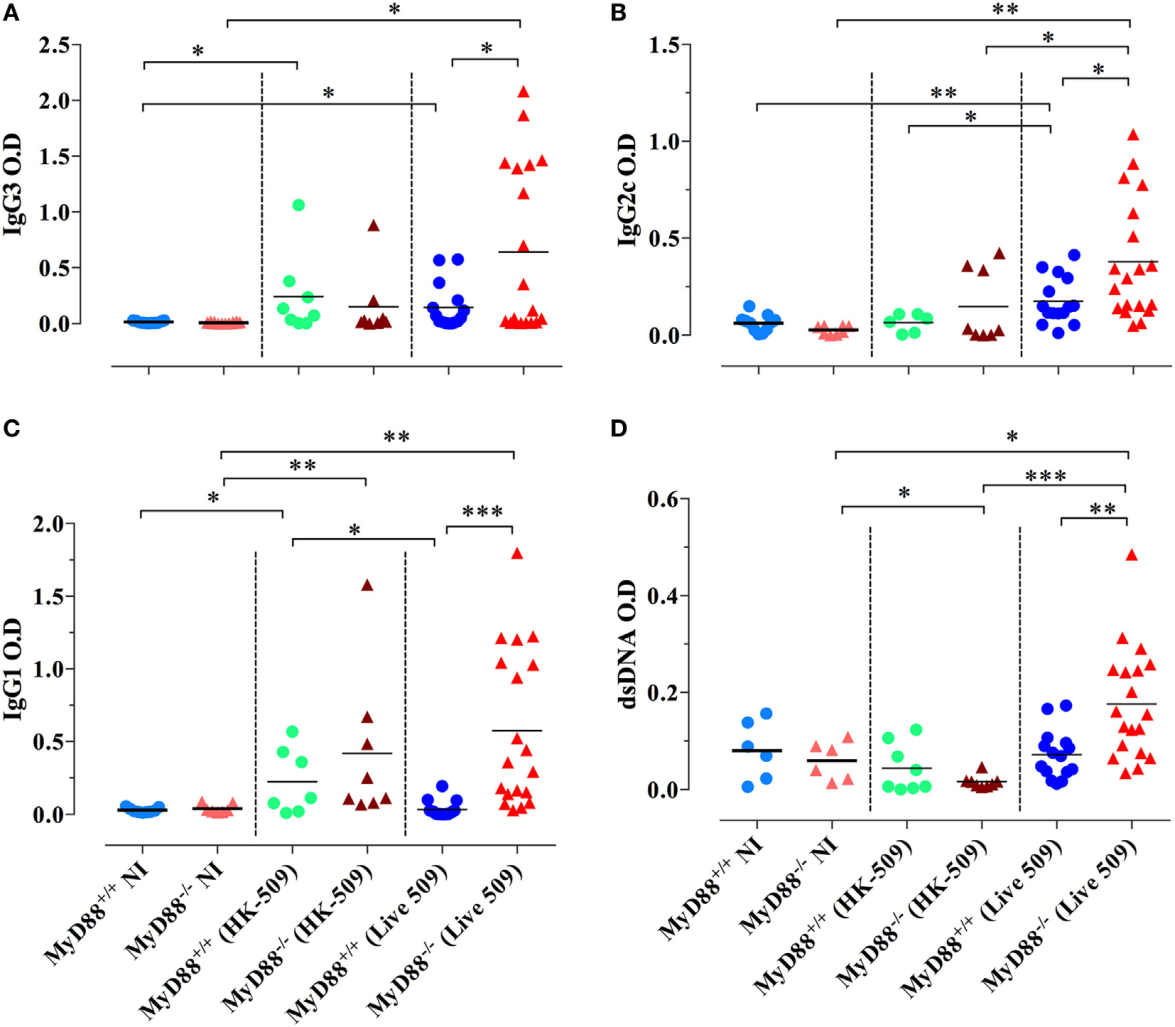
Absence of immunoglobulin dysregulation and autoantibodies in MyD88^−/−^ mice following injection of heat-killed (HK) BRD509. Serum levels of *Salmonella*-specific IgG3 **(A)**, IgG2c **(B)**, and IgG1 **(C)** antibodies were determined at 8 weeks post i.p. inoculation of 2 × 10^5^ HK BRD509. For comparison, antibody production in response to low dose infection with live BRD509E strain, at 3 weeks postinfection, is shown. **(D)** Serum reactivity to double-stranded DNA (dsDNA) in HK vs live *Salmonella*-infected mice. The cutoff for the detection of dsDNA was determined at 1/1,000 dilution. Data are compiled from two independent experiments (*p < 0.05; **p < 0.01; ***p < 0.001).

We also tested the capacity of HK *Salmonella* (strain BRD509E) to induce hypergammaglobulinemia. Although triggering a larger production of IgG1 subclass in MyD88^−/−^ mice in comparison to wild-type mice, the antibody levels in sera were lower than those induced when MyD88^−/−^ mice were infected with live bacteria. HK *Salmonella* was unable to significantly trigger the production of IgG3 and IgG2 subclasses (Figures 10A–C) as well as ANA antibodies in both wild-type and MyD88^−/−^ mice (Figure 10D). These results indicate that the nature of *Salmonella* antigens is critical for the breakdown in B cell self-tolerance in MyD88^−/−^ mice.

## Discussion

After penetration of epithelial barrier through M cells overlying the lymphoid follicles of Peyer’s patches, *S. typhimurium*, which is an important model commonly used to study human immune responses to *S. typhi* (58), typically infect macrophages, causing a self-limiting infection in the lamina propria. However, an impaired immune response can result in uncontrolled spread of bacteria into deeper organs such as spleen and liver, where the immune responses try to control the massive bacterial proliferation (22, 59, 60). Here, we show that even low doses (~2 × 10^2^ CFUs/mouse) of an attenuated strain of *S. typhimurium* (29, 31) can cause a disseminated disease in MyD88^−/−^ mice, as detected by shedding of bacteria in their stools, splenomegaly, and significantly elevated bacterial burden in spleen and liver compared to MyD88^+/+^ mice. Our data are in agreement with Seibert et al. (22) who reported a defective control of bacterial spread in response to the attenuated *Aro*^−^ strain SL7207 and Ko et al. (21) who demonstrated that MyD88-dependent innate immune responses are indispensable for protection against another attenuated strain of *S. typhimurium*. Taken together, the data described above suggest that despite the well-characterized phenotypic differences between MyD88-deficient mice and humans, both species are naturally highly susceptible to invasive *Salmonella* infections (16, 17). Importantly, different attenuated bacteria have been designed to effectively work as live vaccines by inducing protection against invasive *Salmonella* diseases (61). Moreover, recombinant strains of *Salmonella* engineered to express immunomodulatory cytokines have been shown to boost innate and adaptive immune responses in normal as well as in immunodeficient hosts (13, 18, 29, 33, 62, 63). The success of these preclinical studies promises to improve the public-health problems caused by *Salmonella* infections, such as those caused by multidrug-resistant strains (64–69) and the high mortality of infected children (70, 71). However, adverse side effects of live vaccines in MyD88-deficient patients have been reported (72). Thereby, the translation of *Salmonella* live vaccines to the clinical routine will need to be carefully monitored to avoid severe adverse effects in infants with inborn errors of TLR signaling.

The establishment of persistent infections by bacterial species, such as *S. typhimurium*, is associated with considerable tissue damage, leading to hyperactivation of immune responses (28). In this context, the characterization of dysregulated adaptive immune responses, including hypergammaglobulinemia, in MyD88^−/−^ mice remains to be explored. Seibert et al. (22) reported a reduced accumulation of CD11b^+^Gr1^+^ myeloid cells in spleens of infected MyD88^−/−^ mice, which are unable to clear *S. typhimurium* (SL7207 strain). However, with the exception of elevated *Salmonella*-specific IgG1 antibodies, the authors found normal levels of anti-*Salmonella* IgM, IgG2b, and IgG2c as well as apparently no abnormalities in T cell responses, suggesting a minimal alteration of humoral and cellular immunity against *S. typhimurium*. On the other hand, Ko et al. reported that *S. typhimurium-*infected (RASV strain) MyD88^−/−^ mice display expanded numbers of B cells, CD4^+^ T cells, and CD11b^+^Gr1^+^ myeloid cells in their spleen (73). Furthermore, MyD88^−/−^ mice develop dysregulated antibody responses characterized by increased serum levels of IgG, IgA, and IgM *Salmonella*-specific antibodies, production of anti-dsDNA autoantibodies, and deposition of immune complexes in kidneys in a T_FH_ cell-dependent manner.

The apparent differences between the aforementioned studies could be due to different approaches such as the route of bacterial administration (17) and/or the bacterial strains used (74), thus requiring further investigation. Our present findings indicate that abnormalities of myeloid and T cells from MyD88^−/−^ mice contribute systemically to the development of an abnormal humoral immunity characterized by high serum levels of *Salmonella*-specific IgM, IgG3, IgG2c, and IgG1 antibodies and enhanced levels of anti-dsDNA, anti-thyroglobulin, and IgG RF. Our data also suggest that the *Salmonella*-driven B cell activation (73, 75) is dependent upon the establishment of a chronic infection, since only live bacteria were able to induce hypergammaglobulinemia and autoantibody production in MyD88^−/−^ mice. Interestingly, the dysregulation of humoral immune responses in MyD88^−/−^ mice seem to be dependent upon the species of pathogen that mice are challenged with. Thus, while MyD88^−/−^ mice infected with different attenuated *Salmonella* strains or with *B. burgdorferi* (23) developed extreme hypergammaglobulinemia compared to MyD88^+/+^ animals, other bacterial species (*A. baumanii, S. agalactiae*, and *E. coli*) were unable to trigger the same phenomenon. Therefore, our findings provide an important link between environmental factor, genetic background, and the potential development of autoimmune diseases.

The hyperactivation of B cell responses in the context of a chronic infection in MyD88^−/−^ mice correlates with aberrant activation of myeloid cells and Tfh cells and the production of BAFF cytokine. Increased levels of BAFF breach B cell tolerance by enhancing survival of self-reactive B cells, thus allowing their abnormal entry into the mature follicular compartment where they can receive T cell help (76). A suppressive role for B cell-intrinsic MyD88 expression has also been proposed (77), which may well underlie the breakdown of B cell self-tolerance in our *Salmonella* infection model. Our current findings show that the autoantibody levels produced in chronically infected MyD88^−/−^ mice are comparable to, or higher than, those in autoimmune MRL-lpr mice. An inflammatory positive-feedback loop involving activated BAFF-producing myeloid cells and IFNγ-secreting T cells has been shown to play an important role in driving autoantibody production in autoimmune-prone mice (78). Purified T cells of infected MyD88^−/−^ mice exhibited a heterogeneous cytokine expression pattern, including IL-21, IL-4, BAFF, IL-10, and IFN-γ and upregulated PD-1. Moreover, intracellular cytokine staining confirmed the increased presence of splenic T cells co-expressing IFN-γ plus IL-4 or IFN-γ plus IL-10 in *Salmonella*-infected MyD88^−/−^ mice. This heterogeneous cytokine expression profile is typical of Tfh cells (51). Collectively, in the absence of MyD88, a persistent infection induces hypergammaglobulinemia and autoantibody development driven by the aberrant activation of myeloid cells and BAFF secretion. This, in turn, activates Tfh cells which consequently drive aberrant B cell responses.

In an attempt to translate our findings to humans, during approximately 10 years after the first report suggesting that the MyD88 signaling pathway is essential for the removal of autoreactive B cells, paradoxically neither autoreactive antibodies were identified in the serum of MyD88-deficient patients nor these subjects were reported to develop autoimmune diseases (72, 79). However, very few MyD88-deficient patients have been characterized so far (72). The clinical spectrum of MyD88-deficiency remains to be determined in different geographic regions of the world where MyD88-deficient subjects will enter in contact with different pathogens that could trigger the development of self-reactive B cells. In line with this hypothesis, during the time of manuscript preparation, a single Myd88-deficient patient and four with IRAK4 deficiency were reported to develop a particular pattern of self-reactive B cells, which are expanded during lupus flares, and speculated to be modulated by alterations in human microbiome (80). This indicates that patients with MyD88 deficiency must be monitored for the development of autoreactive B cells and that improvement in the genetic diagnosis of this primary immunodeficiency worldwide could provide a priceless opportunity to uncover new MyD88-dependent mechanisms that orchestrate the adaptive immune response.

In conclusion, our findings expand the knowledge about the role of the TLR-MyD88 pathway in the host protection against *Salmonella* infections. Importantly, these data further characterize the dysregulation of adaptive immune system triggered by *S. typhimurium* in MyD88^−/−^ mice. Considering the link between *Salmonella* infections and the development of autoimmune diseases (26, 27, 81, 82), future studies to further investigate the nature of immune dysregulation induced by *Salmonella* species in immunocompromised and immunocompetent hosts could reveal several novel important immunological mechanisms that can be explored as new therapeutic targets.

## Ethics Statement

All studies involving animals were conducted in accordance with and after approval of the animal research ethics committee of the College of Medicine and Health Sciences, United Arab Emirates University.

## Author Contributions

JI performed experiments and analyzed data. YM provided valuable suggestions and support for all molecular studies. GB provided valuable support for histological studies. AA-S performed ELISA experiments and analyzed data. WC contributed to the design of the study. TK and AI performed bioinformatics analysis. GR contributed to the discussion. KB and RL provided valuable reagents. OC-M contributed to data analysis and interpretation and to manuscript writing. MF-C supervised the project and wrote the final manuscript. Ba-R designed the study, supervised the project, analyzed data, and wrote the final manuscript. All authors read and approved the final manuscript.

## Conflict of Interest Statement

The authors declare that the research was conducted in the absence of any commercial or financial relationships that could be construed as a potential conflict of interest. The reviewer TW and the handling Editor declared their shared affiliation.
