# Supplementary material for: Induction of Hypergammaglobulinemia and Autoantibodies by Salmonella Infection in MyD88-Deficient Mice
Source: Front Immunol. 2018 Jun 20;9:1384. doi: 10.3389/fimmu.2018.01384 (PMC6019449; doi:10.3389/fimmu.2018.01384)
Supplement: Supplementary file 1 [file Presentation_1.PDF]

Induction of hypergammaglobulinemia and autoantibodies by Salmonella infection in MyD88-deficient mice

Jincy M. Issac, Yassir A. Mohamed, Ghada Bashir, Ashraf Al-Sbiei, Walter Conca, Taj A. Khan, Asif Iqbal, Gabriela Riemekasten, Katja Bieber, Ralf J. Ludwig, Otavio Cabral-Marques, Maria J. Fernandez-Cabezudo, and Basel K. al-Ramadi

SUPPLEMENTARY DATA

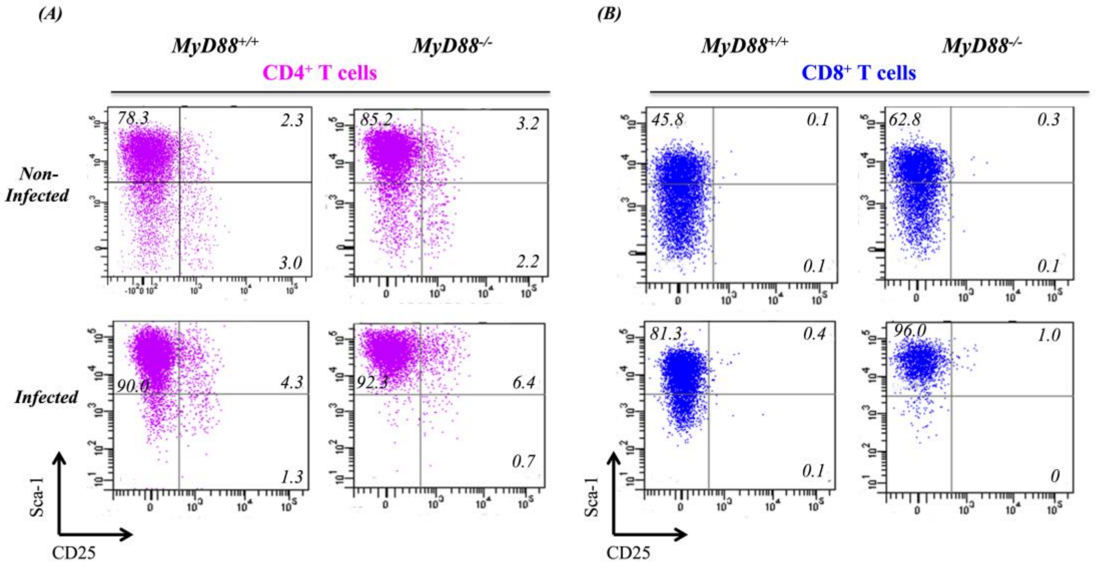

Supplementary Fig. 1. Increased T cell activation in the spleens of MyD88$^{-/-}$ mice following infection with Salmonella. Expression of Sca-1 and CD25 proteins on gated CD4^{+} (*A*) and CD8+ (*B*) T cells from the indicated mouse strains is shown. Percent of various cell populations is shown in each quadrant. The data are representative of three independent experiments.

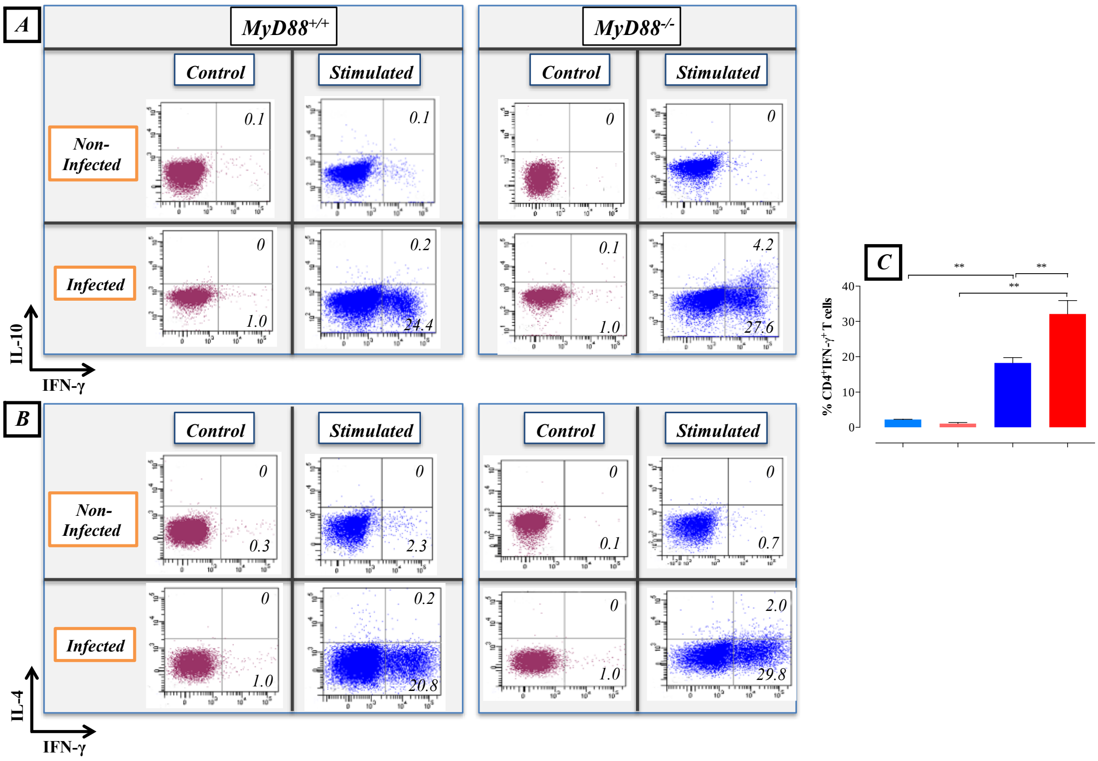

Supplementary Fig. 2. Co-production of IFN-γ and IL-10 or IFN-γ and IL-4 by CD4$^+$ T cells of MyD88$^{-/-}$ mice following Salmonella infection. CD4$^+$ T cells were purified from non-infected or Salmonella-infected spleens of WT and MyD88$^{-/-}$ mice at day 21 post infection. Purified CD4$^+$ T cells were cultured with or without overnight stimulation with plate bound anti-CD3/CD28 antibodies and analyzed. Cytokine secretion by purified T cells was detected by intracellular staining for IFN-γ and IL-10 (***A***) or IFN-γ and IL-4 (***B***). Percent of various cell populations is shown in each quadrant. The data are representative of three independent experiments. (***C***) Cumulative data showing the percent of CD4$^+$ T cells expressing IFN-γ in the different experimental groups. All determinations were done in triplicates (*, p<0.05, **, p<0.01).

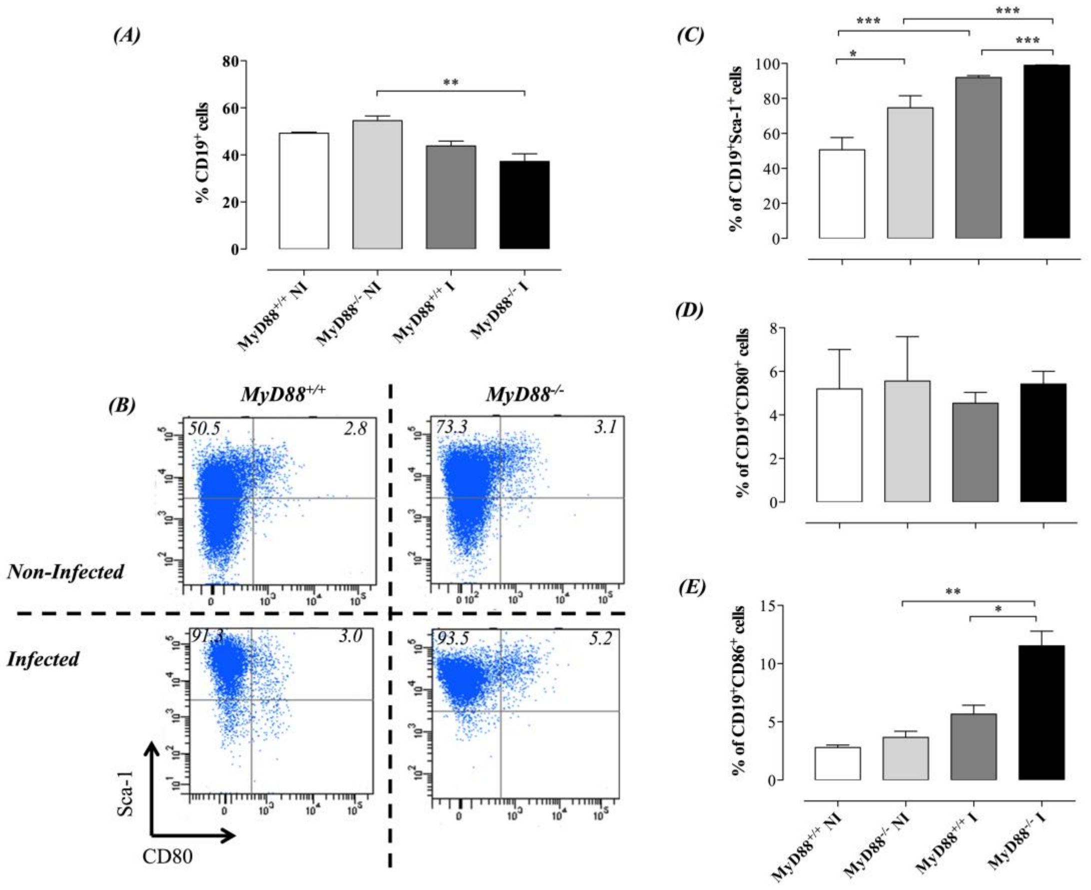

Supplementary Fig. 3. Increased B lymphocyte activation in the spleens of MyD88$^{-/-}$ mice following infection with *Salmonella.* **(A)** Percentage of CD19^{+} B cells in infected and non-infected groups. **(B)** Dot plots depicting the level of expression of Sca-1 and CD80 proteins on gated CD19^{+} B cells in non-infected and Salmonella infected MyD88$^{+/+}$ or MyD88$^{-/-}$ mice. Numbers within quadrants represent percentage of positive cells in that population. Results of individual mice are shown and are representative of four independent experiments. (**C-E**). Percentages of CD19^{+} lymphocytes expressing Sca-1 (***C***), CD80 (***D***) or CD86 (***E***) proteins in non-infected or BRD509-infected MyD88$^{+/+}$ or MyD88$^{-/-}$ mice. Graphs are compiled from 4 independent experiments (*, p<0.05, **, p<0.01, ***, p<0.001).

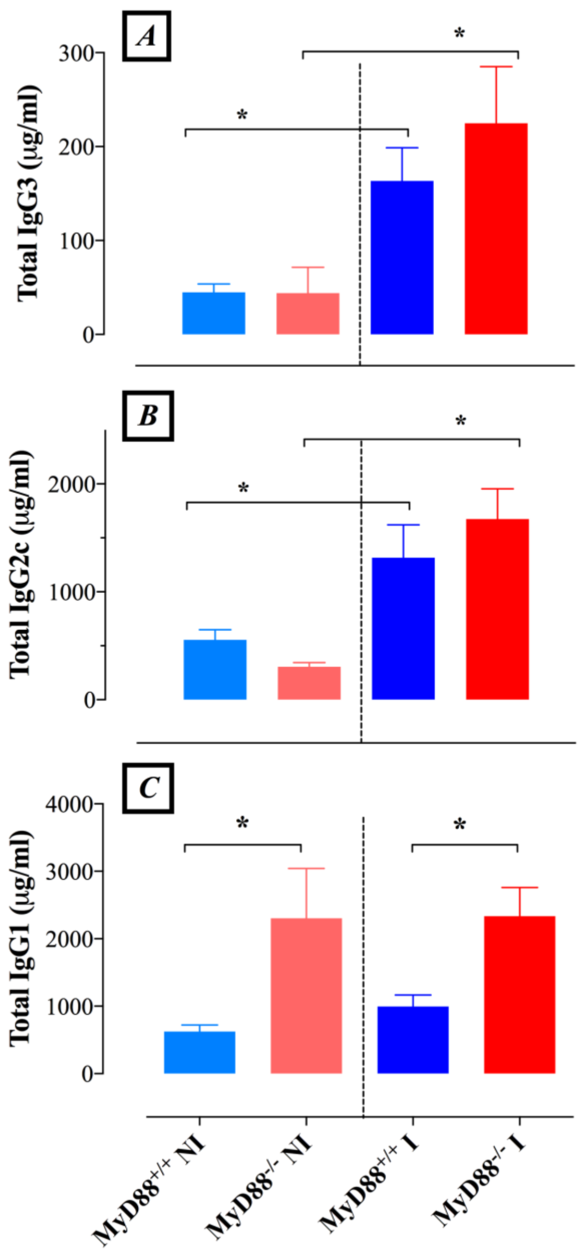

Supplementary Fig. 4. Quantification of total serum antibodies in MyD88-deficient mice. Following infection with BRD509 (~200 CFUs/mouse), sera were collected 4-6 weeks later. Total serum IgG3 (***A***), IgG2c (***B***), and IgG1 (***C***) in both infected and non-infected wild-type and MyD88$^{-/-}$ mice were quantified by ELISA. Each data point represents mean ± SEM of 3–10 mice per group (*, p<0.05).
